# Bioactive Compounds in *Citrus reticulata* Peel Are Potential Candidates for Alleviating Physical Fatigue through a Triad Approach of Network Pharmacology, Molecular Docking, and Molecular Dynamics Modeling

**DOI:** 10.3390/nu16121934

**Published:** 2024-06-18

**Authors:** Amin Ullah, Qiuxi Sun, Jiangtao Li, Jinjie Li, Pipasha Khatun, Guangning Kou, Quanjun Lyu

**Affiliations:** 1 Department of Nutrition and Food Hygiene, School of Public Health, Zhengzhou University, Zhengzhou 450001, China; 2 Centre for Nutritional Ecology and Centre for Sport Nutrition and Health, Zhengzhou University, Zhengzhou 450001, China; 3 Department of Public Health, Zhengzhou Shuqing Medical College, Zhengzhou 450001, China

**Keywords:** network pharmacology, physical fatigue, molecular docking, *Citrus reticulata* peel, molecular dynamics simulation

## Abstract

Physical fatigue (peripheral fatigue), which affects a considerable portion of the world population, is a decline in the ability of muscle fibers to contract effectively due to alterations in the regulatory processes of muscle action potentials. However, it lacks an efficacious therapeutic intervention. The present study explored bioactive compounds and the mechanism of action of *Citrus reticulata* peel (CR-P) in treating physical fatigue by utilizing network pharmacology (NP), molecular docking, and simulation-based molecular dynamics (MD). The bioactive ingredients of CR-P and prospective targets of CR-P and physical fatigue were obtained from various databases. A PPI network was generated by the STRING database, while the key overlapping targets were analyzed for enrichment by adopting KEGG and GO. The binding affinities of bioactive ingredients to the hub targets were determined by molecular docking. The results were further validated by MD simulation. Five bioactive compounds were screened, and 56 key overlapping targets were identified for CR-P and physical fatigue, whereas the hub targets with a greater degree in the PPI network were AKT1, TP53, STAT3, MTOR, KRAS, HRAS, JAK2, IL6, EGFR, and ESR1. The findings of the enrichment analysis indicated significant enrichment of the targets in three key signaling pathways, namely PI3K-AKT, MAPK, and JAK-STAT. The molecular docking and MD simulation results revealed that the bioactive compounds of CR-P exhibit a stronger affinity for interacting with the hub targets. The present work suggests that bioactive compounds of CR-P, specifically Hesperetin and Sitosterol, may ameliorate physical fatigue via the PI3K-AKT signaling pathway by targeting AKT1, KRAS, and MTOR proteins.

## 1. Introduction

Fatigue, which originates from the Latin word fatigare, referring to “to tire,” is characterized as extreme tiredness caused by physical or mental exertion or disease [1]. Fatigue can be central (mental) or peripheral (physical), both of which affect more than 20% of the global population [2]. McKenna et al. were among the first authors to explain that fatigue can be caused by two main factors: central fatigue, which arises from the central nervous system, and peripheral fatigue, which involves the muscles and originates from the peripheral nervous system. Peripheral fatigue, also known as physical fatigue, refers to a decrease in the ability of muscle fibers to contract efficiently. This decrease is caused by alterations in the processes that control muscle action potentials [3]. Central fatigue (mental fatigue) refers to a decrease in an individual’s ability to perform cognitive tasks requiring focus and attentiveness [4]. Millions of individuals encounter fatigue and its associated disorders due to the escalating pace of life and social competition in contemporary society. Fatigue is a multifaceted physiological and biological occurrence characterized by mental or physical energy depletion. It is characterized by a severe lack of energy or exhaustion, and it has numerous detrimental effects on individuals’ productivity and quality of life [5]. Long-term persistent fatigue can negatively affect an individual’s physical and mental well-being, as well as lay a foundation for various diseases, including depression, chronic fatigue syndrome, hypertension, coronary heart disease, cancer, and karoshi [6]. Peripheral fatigue has been suggested to be significantly influenced by a reduction in glycemic levels and tissue glycogen content in addition to an excess of metabolites, including lactate and urea nitrogen production and accumulation [2]. Furthermore, excessive reactive oxygen species (ROS) can be produced in muscles and other organs, including the liver, during exercise and stress. These ROS can induce lipid peroxidation and cellular metabolism disruption, causing damage to skeletal muscle and liver mitochondria. Consequently, such damage can lead to physical fatigue [5]. 

A novel concept in modern drug research, herbal medicines, particularly natural drugs, provide a vast resource for screening natural products with active and less toxic adverse effects [7]. Considerable attention has been drawn towards natural products because of their potential health benefits and antioxidant characteristics. Specifically, grapefruits, mandarins, oranges, lemons, and limes are all prominent fruits classified within the citrus genus, a constituent of the Rutaceae family [8]. One such natural product is *Citri Reticulata* peel (CR-P), a dried pericarp of the mature fruit of *Citrus reticulata* Blanco, and it belongs to the Rutaceae family and the citrus genus. It is a globally distributed product commonly known as tangerine peel, referred to as Chenpi in Chinese [9,10,11]. Prominent bioactive compounds in CR-P, as identified by previous studies, are flavonoids, which consist of Hesperidin, Hesperetin, Naringenin, Nobiletin, Naringin, Tangeretin, and Erodcyol [7]. These bioactive compounds possess various health benefits, including anti-inflammatory, antioxidative, antimutagenic, anticancer, anti-microbial, anti-viral, anti-diabetic, and anti-glycemic activities [12]. CR-P, a herb with significant cholesterol-lowering and weight-loss-promoting properties, has been extensively utilized in traditional Chinese medicine (TCM) for years to treat several diseases [13]. However, the current scientific literature lacks a clear understanding of whether CR-P has a role in alleviating physical fatigue. 

Physical fatigue presently lacks an efficacious therapeutic intervention. The potential function of bioactive compounds in natural products as anti-fatigue agents has been suggested by numerous studies that demonstrate their ability to prevent and alleviate physical fatigue safely and effectively [14]. However, the underlying mechanisms, targets, and pathways by which these natural compounds may alleviate physical fatigue need to be explored. The application of network pharmacology and bioinformatic approaches to find novel therapeutic agents and investigate the therapeutic mechanisms of diseases has attracted considerable attention in contemporary research owing to the ongoing progress in the databases of diverse drugs and diseases. Facilitating the comprehension of the pharmacology of drugs and their impacts on biological networks, network pharmacology (NP) is a systematic approach to identifying relationships between targets, diseases, and drugs [15]. An emerging interdisciplinary field, NP has been utilized to analyze the functional mechanisms of TCM. Additionally, it is employed to unveil the active constituents of natural bioactive compounds for treating various diseases [16]. NP endeavors to construct a multi-tiered network utilizing diverse methodologies, such as database retrieval and high-throughput omics data analysis. The overarching objective is to yield profound insights and a scientific foundation for exploring TCM, facilitating an enhanced understanding of the laws and mechanisms governing drug-body interactions at the biomolecular level. The implementation of network pharmacology introduces novel perspectives for the systematic investigation of TCM. Molecular docking, a technique rooted in the properties of receptors and the interactions between drug molecules and receptors, has become pivotal in computer-aided drug discovery. Its ability to accurately predict the binding mechanisms and affinities of molecular interactions positions it as a fundamental method in contemporary drug development endeavors [17]. Therefore, in this study, we utilized a combination of network pharmacology along with molecular docking and molecular dynamics modeling to investigate the bioactive compounds, potential targets, and molecular mechanisms of CR-P in treating physical fatigue.

## 2. Materials and Methods

### 2.1. Establishing Database for Bioactive Compounds of Citrus reticulata Peel 

PubChem^®^, a chemical database maintained by the National Library of Medicine, is widely used for drug repurposing and virtual screening. It is publicly accessible at https://pubchem.ncbi.nlm.nih.gov (accessed on 10 February 2024) [18]. A database for five bioactive compounds of *Citrus reticulata* peel (CR-P) was established. The canonical SMILES and three-dimensional (3D) structures of these compounds were acquired from the PubChem database by using the keywords “Sitosterol”, “Naringenin”, “Hesperetin”, “Citromitin”, and “Nobiletin”, respectively. The best matches for these compounds were selected, and 3D conformers were downloaded and saved as SDF files for further analysis. The Sitosterol, Naringenin, Hesperetin, Citromitin, and Nobiletin of CR-P were used as the main compounds against physical fatigue in this study. 

### 2.2. Identification of Bioactive Compounds of CR-P and Related Targets

The potential targets and bioactive compounds of CR-P were screened and collected by a comprehensive search in Traditional Chinese Medicine Systems Pharmacology (TCMP) database (https://tcmsp-e.com/) (accessed on 10 February 2024). The TCMSP database is an interactive information resource that serves as a systematic pharmacology platform for Chinese herbal medicine. It offers information regarding the interactions of drugs, targets, and diseases [19]. The database comprises 837 related diseases, 12,144 compounds, and 499 Chinese herbal remedies registered in the Chinese Pharmacopoeia. The entries can be filtered by users based on pharmacokinetic parameters, which include drug absorption, distribution, metabolism, and excretion (ADME) [20]. The bioactive ingredients were screened in the TCMP database using the keywords “Citrus reticulata” or “Chenpi” as herbal names. In order to evaluate bioactive compounds, the oral bioavailability (OB) and drug-likeness (DL) criteria were established at ≥30% and ≥0.18, respectively [21]. Drug utilization efficiency is represented by OB, which is the percentage of intact drugs that enter the circulatory system following oral administration, whereas DL refers to the capacity of a compound to be classified as a drug [22]. Following screening by OB and DL criteria, the related targets of these bioactive compounds were identified in the database. In addition, another comprehensive search was carried out in the BATMAN-TCM (http://bionet.ncpsb.org.cn/batman-tcm/) (accessed on 11 February 2024), which is an annotation tool of bioinformatics designed to annotate the molecular mechanisms of TCM. For evaluating the effect targets of bioactive compounds, the BATMAN-TCM database is extensively utilized in TCM. A druggable score of ≥0.1 and a confidence score of ≥0.95 were set as screening criteria in the BATMAN-TCM database. Inclusion was restricted to specific targets that functioned as complementary factors to achieve the targets with effective chemical composition [23]. Furthermore, more targets for these bioactive compounds were identified in the PharmMapper database (https://www.lilab-ecust.cn/pharmmapper/submitfile.html) (accessed on 16 February 2024) [24]. The PharmMapper database is supported by an extensive internal registry of pharmacophore databases comprised of all targets in DrugBank, TargetBank, BindingDB, and PDTD [25]. The prediction database (https://prediction.charite.de/) (accessed on 16 February 2024) was used to retrieve more predicted targets by putting the canonical SMILES of bioactive compounds into the search engine of the database [17]. The target gene names were obtained by importing the target protein names into the UniProtKB module of the UniProt database (https://www.uniprot.org) (accessed on 18 February 2024) [26,27], with a search filter of “Human species” and “Reviewed”. Subsequently, targets from these databases were combined, and any duplicates were eliminated.

### 2.3. Identification of Physical Fatigue-Related Targets

Potential targets of physical fatigue were retrieved by a keyword search of “physical fatigue” in the GeneCards database (https://www.genecards.org/) (accessed on 20 February 2024). The potential targets of physical fatigue were screened by using a relevance score of ≥10, and the results were saved for further analysis [28]. Additionally, potential targets of physical fatigue were acquired by a keyword “physical fatigue” search with the Gene Map filter in the Online Mendelian Inheritance in Man (OMIM) database (https://www.omim.org/) (accessed on 20 February 2024) [29]. Targets with approved symbols were selected for further analysis. The retrieved targets were subsequently merged and the duplicates were removed.

### 2.4. Key Overlapping Targets of CR-P and Physical Fatigue

To find the key overlapping targets between drug and disease, the potential targets of bioactive compounds of CR-P and physical fatigue were imported into the (http://www.interactivenn.net/) online platform (accessed on 21 February 2024) [15], and the Venn diagram was constructed.

### 2.5. Network Construction of Protein–Protein Interaction (PPI)

The PPI network was established by importing the key overlapping targets of CR-P and physical fatigue into the STRING database version 12.0 (https://string-db.org/) (accessed on 21 February 2024). An online search was performed in the STRING platform with a search filter set as “Multiple proteins” and organism as “Homo sapiens”. The system setting was set as the minimum interaction score with a medium confidence of “0.400”, while “hide disconnecting nodes in the network” was also selected [27,30]. The remaining settings were left as the system default. The constructed PPI network was exported as a TSV file and saved for further analysis. The network was then analyzed and visualized by Cytoscape 3.9.1. The top ten hub genes were calculated by utilizing the cytoHubba plugin tool of the Cytoscape software. The selection of the parameters for this calculation was predicated on the maximal clique centrality (MCC) of the top ten nodes.

### 2.6. GO and KEGG Pathway Enrichment Analysis

The key overlapping targets generated by (http://www.interactivenn.net/) (accessed on 21 February 2024) were imported into the Metascape database, an online gene annotation and analysis resource platform (https://metascape.org/) (accessed on 24 February 2024). In the era of big data, the Metascape database serves as an effective platform for conducting thorough analyses and interpretations of omics. It offers a variety of functionalities, including gene annotation, interactive analysis, and function enrichment. A macro-level understanding of the functionality of enormous genes can be attained by combining KEGG and GO enrichment analyses and identifying drug-disease signaling pathways [20]. Gene Ontology (GO) and Kyoto Encyclopedia of Genes and Genomes (KEGG) analyses were carried out by using the Metascape database; correspondingly, information was obtained regarding biological process (BP), cellular components (CC), molecular functions (MF), and KEGG pathways. The system setting was set to “gene list” as the list type, and the “*Homo sapiens*” species was selected. A *p* value of less than 0.01, an enrichment factor greater than 1.5, and a minimal overlap of 3 were set for the enrichment analysis. Bar graphs and bubble charts were generated after importing the top 20 KEGG pathways and top 10 GO terms based on their *p* value into an online bioinformatics mapping platform (https://www.bioinformatics.com.cn/en) (accessed on 26 February 2024) [15].

### 2.7. Network Construction of Target-Pathway Interaction

The target-pathway interaction network was generated in Cytoscape 3.9.1 by importing the top 20 KEGG pathways and their related targets, along with the bioactive compounds and CR-P, from the enrichment analysis. Consequently, the target–pathway network of bioactive compounds of CR-P and physical fatigue was visualized by the software and a diagram was drawn.

### 2.8. Molecular Docking

Molecular docking is a technique that can somewhat predict the activity and binding mechanisms of active components with potential target proteins by establishing connections between small molecules and relevant targets [31]. The 3D structures of the bioactive compounds that were intended to interact with target proteins were obtained and saved as SDF files. These structures were downloaded from (https://pubchem.ncbi.nlm.nih.gov/) (accessed on 2 March 2024). The SDF file containing these compounds was subsequently imported into Chem3D software, where the energy minimization operation was performed, and the resulting file was saved in the mol2 format. The UniProt database was utilized to acquire the 3D structure of the potential target proteins. The crystal X-ray structures of the hub targets used by previous studies were acquired from the Worldwide Protein Data Bank (RCSBPDB) database (https://www.rcsb.org/) (accessed on 2 March 2024). Specifically, structures from the “Homo sapiens” species with resolutions between 2.0 and 3.0 Å were selected [32]. Water and ligand molecules were removed from protein structures by PyMol 2.5.8. The protein structures were incorporated into AutoDock Tools 1.5.6, where they were assigned non-polar hydrogens and transformed into the PDBQT format. Furthermore, for the purpose of docking, the small molecule ligands (Sitosterol, Naringenin, Hesperetin, Citromitin, and Nobiletin) were transformed into the PDBQT format. Finally, docking was performed by Autodock Vina software with lower binding energy, indicating a more stable structure. The binding of bioactive compounds with the hub targets was visualized by PyMol 2.5.8, and the docking diagram and pocket diagram were drawn. PyMOL is an open-source visualization software for molecular modeling that can identify hydrogen bonds and analyze the binding state of receptor proteins and ligands during molecular docking [20].

### 2.9. Molecular Dynamics Simulation

The binding affinities of bioactive compounds and target proteins acquired by molecular docking were analyzed using molecular dynamics (MD) simulation. MD simulation is frequently employed in the drug design process and is crucial in comprehending the dynamic mechanisms and conformational alterations of proteins [33]. The simulation was performed by using Amber18 software [34]. In order to parameterize the small molecules, the ANTECHAMBER module and GAUSSIAN 09 software were used [35,36]. For proteins, the ff14SB force field parameters were utilized, whereas, for bioactive compounds, the gaff2 generic force field parameters were employed [37,38]. The LEaP module was utilized to incorporate sodium ions and hydrogen atoms into protein–small molecule complexes in order to achieve charge neutrality in the system. The periodic boundary parameters were established, and the TIP3P water model was chosen [39]. The workflow for molecular dynamics modeling involves four essential steps: energy minimization, heating, equilibration, and simulation of production dynamics. The system’s energy was initially minimized using the steepest descent method for 2500 steps. Subsequently, the conjugate gradient method was employed for an additional 2500 steps. After performing energy minimization, the system was gradually heated to a temperature of 298.15 K over a period of 200 ps. After the heating phase, equilibrium of the system was achieved by conducting NVT (isothermal body) and NPT (isothermal and isobaric) ensemble simulations at 298.15 K for 500 ps. Subsequently, using the NPT ensemble with a 2 fs time step, the system underwent an MD simulation for 100 ns. Data on the trajectory were recorded at intervals of 10 ps. The SHAKE algorithm was implemented to restrain hydrogen atom bonds [40]. The simulation employed the particle mesh Ewald (PME) method to compute the long-range electrostatic interactions. A non-bonded cutoff distance of 10 Å was set. The temperature was controlled by using the Langevin algorithm [41,42]. 

Moreover, the binding free energies between bioactive compounds and proteins were computed by employing the molecular mechanics/generalized born surface area (MM/GBSA) approach. The computation utilized the MMPBSA.py module [43,44,45]. The current work adopted a molecular dynamics trajectory of 90–100 ns for calculation by using the following formula:

$$\begin{array}{ll} {\Delta G}_{bind} & ={\Delta G}_{complex}-({\Delta G}_{receptor}+{\Delta G}_{ligand})\\ & ={\Delta E}_{internal}+{\Delta E}_{VDW}+{\Delta E}_{elec}{+\Delta G}_{GB}+{\Delta G}_{SA} \end{array}$$

${\Delta E}_{internal}$: internal energy

${\Delta E}_{VDW}$: van der Waals interaction

${\Delta E}_{elec}$: electrostatic interactions

${\Delta G}_{GB}$, ${\Delta G}_{SA}$: polar and non-polar solvation free energies 

## 3. Results

The mechanisms of the bioactive compounds of *Citrus reticulata* peel (CR-P) against physical fatigue were investigated using network pharmacology (NP) analysis. This analysis was followed by verification methods such as protein–protein interaction (PPI) networks, Gene Ontology (GO) analysis, Kyoto Encyclopedia of Genes and Genomes (KEGG) pathway enrichment analysis, target–pathway network generation, molecular docking, and molecular dynamics (MD) modeling. 

### 3.1. Bioactive Compounds of CR-P and Related Targets

**Figure 1:**
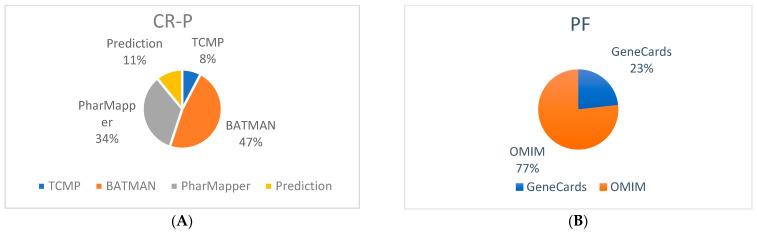
Targets collection from different databases. (**A**) Bioactive compounds related targets of CR-P; (**B**) physical fatigue-related targets.

Five bioactive compounds with 62 targets were retrieved from the TCMP database. In addition, more targets of these compounds were identified in the BATMAN, PharmMapper, and Prediction databases. The retrieved identified targets in the BATMAN, PharMapper, and Prediction database were 382, 274, and 88, respectively (Figure 1A). The targets from these databases were merged. Following the removal of 131 duplicates, 675 potential targets were finalized. Characteristics of bioactive compounds in CR-P are shown in Table 1. 

### 3.2. Potential Targets of Physical Fatigue

Potential targets of physical fatigue were screened in the GeneCards and Online Mendelian Inheritance in Man (OMIM) databases; 100 and 331 targets were retrieved, respectively. The targets were merged, and 147 duplicates were found and removed. A total of 284 potential final targets were obtained for physical fatigue (Figure 1B).

### 3.3. Key Overlapping Targets of CR-P and Physical Fatigue

**Figure 2:**
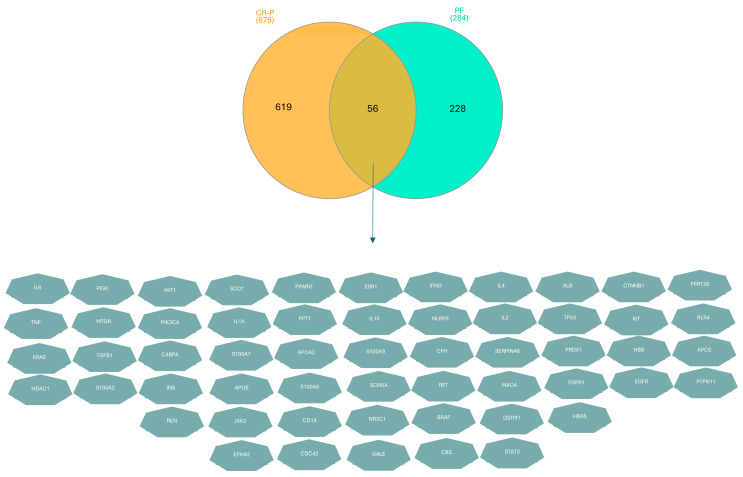
Venn diagram of CR-P and physical fatigue: the gold part represents CR-P with 619 potential targets, the green part represents physical fatigue with 228 potential targets, whereas the tan overlapping part represents 56 common targets between them.

A total of 56 potential target genes were overlapped by the Venn diagram, as shown in Figure 2. These key target genes may have a potential role in alleviating physical fatigue.

### 3.4. Key Overlapping Targets and PPI Network Analysis

**Figure 3:**
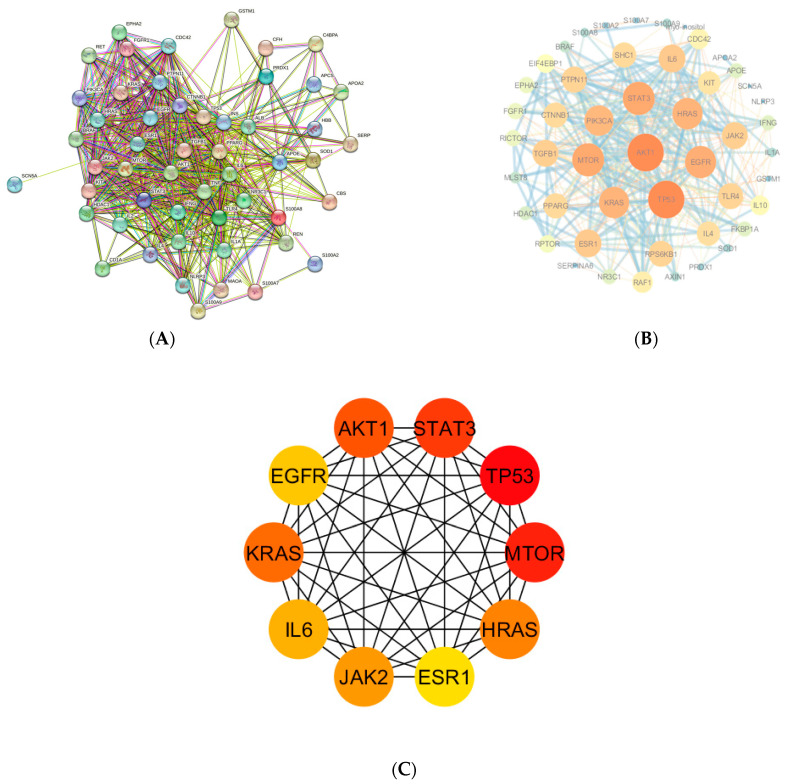
(**A**) PPI network of key targets of CR-P and physical fatigue by STRING database; (**B**) visualized PPI network by Cytoscape; (**C**) hub genes in the PPI network.

The network for PPI was created by incorporating the 56 key overlapping targets into the STRING online platform (Figure 3A). The generated network comprised 368 edges and 55 nodes, with an average node degree of 13.4. Using Cytoscape 3.9.1 software, the generated PPI network was further visualized and analyzed for hub genes. Degree values indicate the magnitude of a node, where larger nodes correspond to greater degrees. The thickness of the line connecting these nodes indicates the level of intersection strength between them (Figure 3B). The computation of the top ten hub genes was performed using the “cytoHubba plugin” within the Cytoscape. AKT1, TP53, STAT3, MTOR, KRAS, HRAS, JAK2, IL6, EGFR, and ESR1 were the top ten hub genes in the network (Figure 3C).

#### GO and KEGG Analysis of Key Overlapping Targets

**Figure 4:**
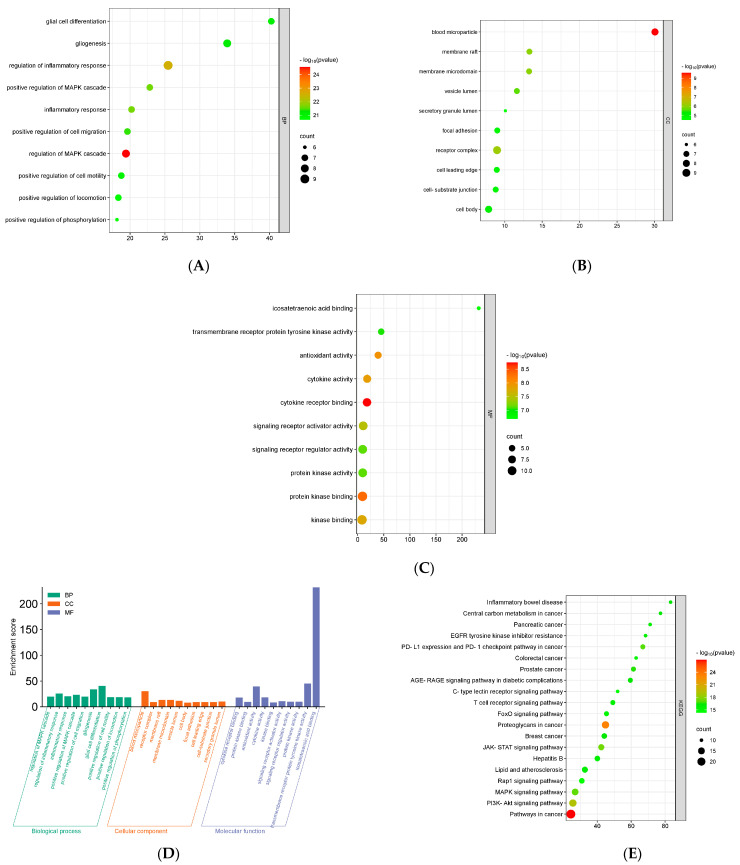
Gene Ontology (GO) and Kyoto Encyclopedia of Genes and Genomes (KEGG) enrichment. (**A**) Biological process; (**B**) cellular component; (**C**) molecular function; (**D**) GO bar chart; (**E**) KEGG enrichment.

GO offers a comprehensive collection of information on the function of genes and gene products, covering biological processes (BPs), cellular components (CC), and molecular functions (MF). On the other hand, KEGG assigns functional importance to genes and genomes at both the molecular and higher levels [46]. The GO of 56 common targets was determined by Metascape with the purpose of identifying the main activities of core targets. A total of 1284 (*p* ≤ 0.01) GO terms were significantly enriched, including 1162 BP, 43 CC, and 79 MF terms. In addition, a substantial enrichment was observed in 153 KEGG pathways. The enrichment results showed the top 10 BP terms were glial cell differentiation, gliogenesis, inflammatory response, regulations of inflammatory response and MAPK cascade, positive regulations of MAPK cascade, cell migration, cell motility, locomotion, and phosphorylation, respectively (Figure 4A). Representative CC terms were blood microparticle, raft membrane, microdomain membrane, vesicle lumen, lumen secretory granule, adhesion of focal, receptor complex, cell leading edge, cell-substrate junction, and cell body (Figure 4B). MF terms included bindings of icosatetraenoic acid, cytokine receptor, protein kinase, and kinase, activities of transmembrane receptor protein tyrosine kinase, antioxidant, cytokine, signaling receptor activator, signaling receptor regulator, and protein kinase, respectively (Figure 4C). The combined GO results can be seen in the bar chart (Figure 4D). 

**Figure 5:**
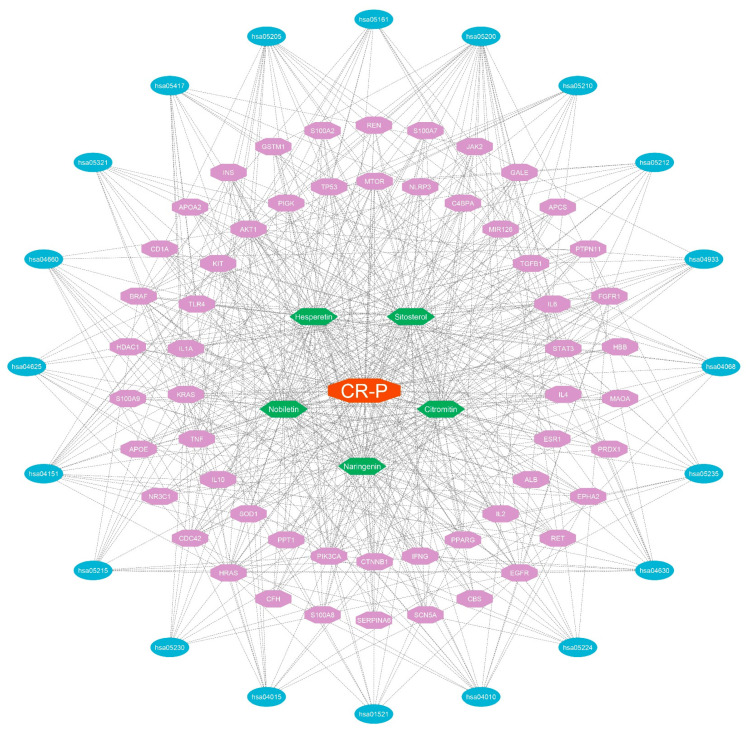
The compound–target–pathway network. The orange octagon in the middle represents CR-P, the green hexagons represent the bioactive compounds, the purple octagons represent the targets, whereas the blue ellipses represent the pathways in the network.

Furthermore, the network’s top 20 KEGG terms were inflammatory bowel disease, central carbon metabolism in cancer, pancreatic cancer, inhibitor resistance of EGFR tyrosine kinase, expression of PD-L1 and PD-1 checkpoint pathway in cancer, colorectal cancer, prostate cancer, proteoglycans in cancer, breast cancer, hepatitis B, lipid and atherosclerosis, and the signaling pathways were AGE-RAGE in diabetic complication, C-type lectin receptor, T cell receptor, FoxO, JAK-STAT, Rap2, MAPK, PI3K-Akt, and pathways in cancer (Figure 4E). The GO and KEGG enrichment results indicate that these targets and pathways may have a substantial impact on the alleviation of physical fatigue by CR-P. The compound–target–pathway network was generated by Cytoscape, as illustrated in (Figure 5). This network utilizes the fact that bioactive compounds in CR-P are capable of acting on multiple targets and pathways, indicating that these compounds may play a significant role in alleviating physical fatigue by aiming at these targets and pathways.

### 3.5. Molecular Docking

**Figure 6:**
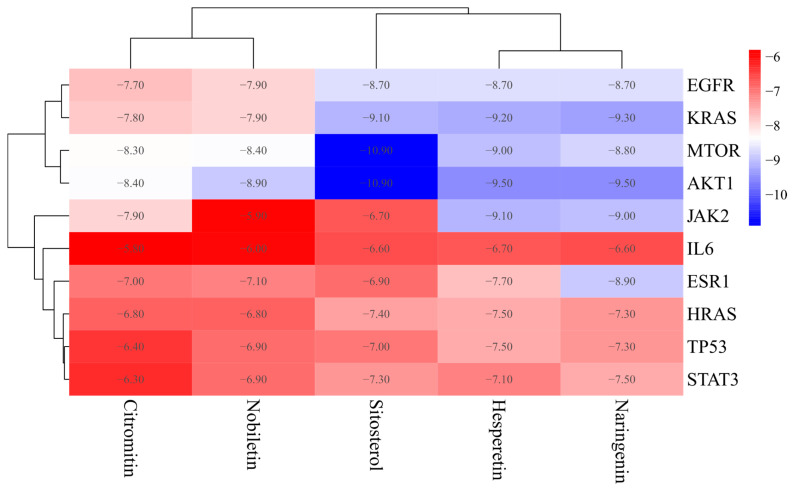
Heatmap of binding affinity scores between bioactive compounds of CR-P and 10 hub targets (kcal/mol) (*n* = 50).

**Figure 7:**
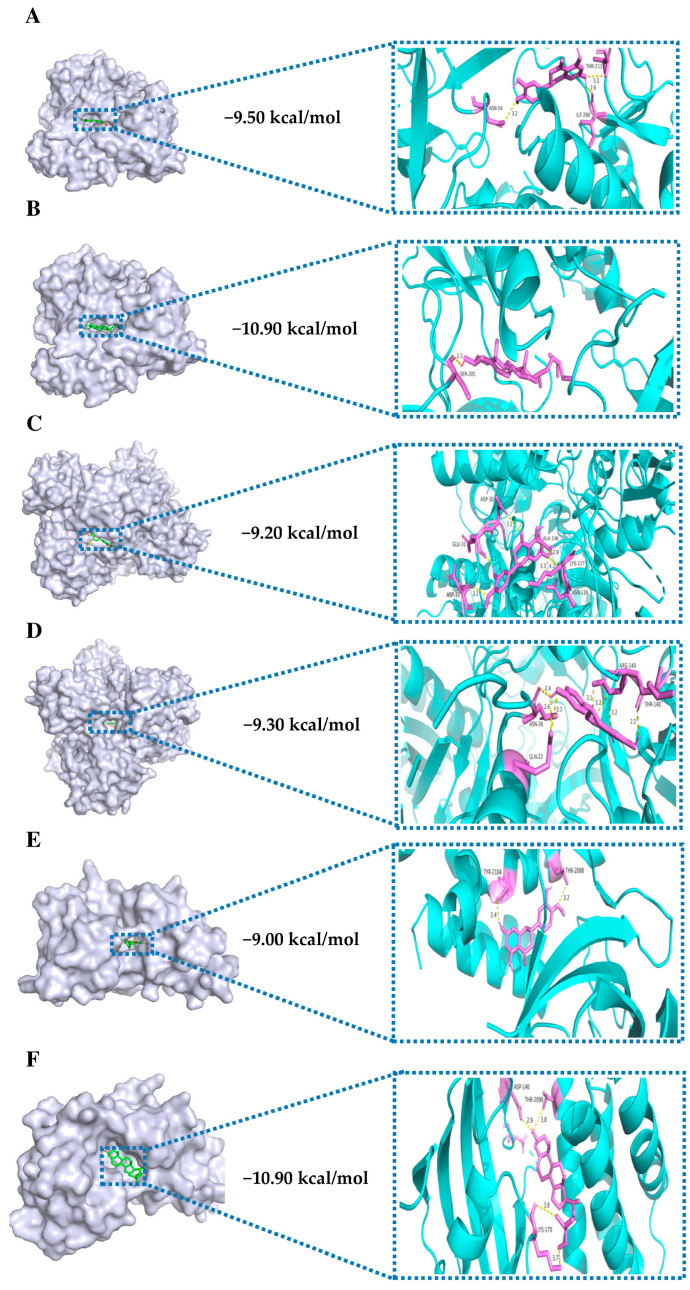
Molecular docking of Hesperetin, Sitosterol, and Naringenin with AKT1, KRAS, and MTOR proteins. (**A**) AKT1–Hesperetin; (**B**) AKT1–Sitosterol, (**C**) KRAS–Hesperetin; (**D**) KRAS–Naringenin; (**E**) MTOR–Hesperetin; (**F**) MTOR–Sitosterol. The cyan color represents proteins, whereas the violet color represents the ligands.

Generally, reduced binding energies lead to more robust interaction probabilities and conformations of binding. According to previous studies, binding energies < 0 kJ/mol indicate spontaneous binding, while values of −5.0 kJ/mol or less suggest favorable binding activity [26]. We performed molecular docking of bioactive compounds, including Sitosterol, Naringenin, Hesperetin, Citromitin, and Nobiletin, with MTOR, TP53, STAT3, AKT1, KRAS, HRAS, JAK2, IL6, EGFR, and ESR1, in order to acquire a more comprehensive understanding of the signaling pathways and interactions between bioactive compounds and hub targets. The maximum and minimum binding energies of these hub targets range from −10.90 to −5.80 kcal/mol, indicating effective binding to these bioactive compounds (Figure 6). The molecular docking analysis revealed that AKT1 had the best binding affinity with these compounds, followed by MTOR, KRAS, JAK2, EGFR, ESR1, HRAS, TP53, STAT3, and IL6. Overall, the compound Sitosterol showed the best docking scores with these hub targets, followed by Hesperetin, Naringenin, Nobiletin, and Citromitin, respectively. The binding performances of the three proteins, namely AKT 1, KRAS, and MTOR, as well as the three bioactive compounds, Hesperetin, Sitostiterol, and Naringenin, exhibited more excellent binding stability in comparison to the other groups (Figure 7). The results indicate that these bioactive compounds bound well to the active sites of these hub targets, implying that these compounds could significantly alleviate physical fatigue via these potential targets.

### 3.6. Molecular Dynamics Modeling

**Figure 8:**
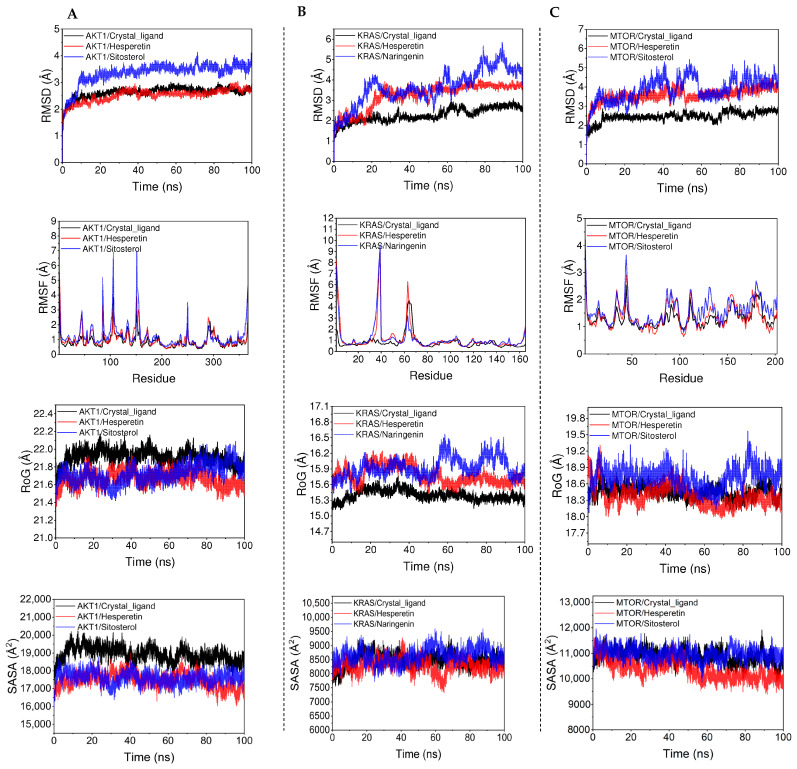
MD simulations of bioactive compounds and target proteins. (**A**) AKT1–Hesperetin/Sitosterol; (**B**) KRAS–Hesperetin/Naringenin; (**C**) MTOR–Hesperetin/Sitosterol.

Based on the findings of molecular docking analysis, three proteins (AKT1, KRAS, and MTOR) and three bioactive compounds (Hesperetin, Sitosterol, and Naringenin), based on their binding interaction, were selected for simulations. The root mean square deviation (RMSD) is a useful indicator for understanding the movement of the complex. The magnitude of the RMSD and the intensity of the fluctuations directly correlate with the magnitude of the motion and vice versa. The AKT1–Crystal_ligand, AKT1–Hesperetin, and AKT1–Sitosterol systems all achieve convergence within the first 10 ns of the simulation and exhibit consistent and stable fluctuations throughout the middle and late stages, indicating stable binding (Figure 8A). The KRAS–Crystal_ligand reaches convergence in the first 10 ns of the simulation, KRAS–Hesperetin reaches convergence at 30 ns, whereas KRAS–Naringenin converges less well. This indicates that KRAS–Crystal_ligand and KRAS–Hesperetin are more stable (Figure 8B). The MTOR–Crystal_ligand and MTOR–Hesperetin reach convergence in the first 10ns of the simulation, while MTOR–Sitosterol converges poorly. It shows that MTOR–Crystal_ligand and MTOR–Hesperetin binding is more stable (Figure 8C). 

Root mean square fluctuation (RMSF) is a useful metric for assessing the protein’s flexibility during MD simulation. Upon binding of a drug to the protein, the protein’s flexibility decreases. This leads to the stabilization of the protein and the activation of the enzyme. The RMSF of most regions of AKT1, KRAS, and MTOR is less than 2 Å, and the rigidity of these proteins is more obvious in the state of binding small molecules. Understanding the radius of gyration (RoG) provides valuable insights into the system’s compactness and overall structure. For the AKT1 protein, the denseness of the protein after Hesperetin and Sitosterol binding is due to the denseness of the protein after crystal ligand binding, indicating the existence of a high binding effect of Hesperetin and Sitosterol on the protein. For KRAS, in regard to the RoG after Hesperetin, the Naringenin binding is larger and the compactness is low, but the overall fluctuation is smoother, implying that there exists a weak binding effect of Hesperetin, Sitosterol, and protein. For MTOR protein, the RoG of the system after Hesperetin binding was comparable to that after crystal ligand binding, indicating that Hesperetin and crystal ligand possessed comparable effects, and the RoG after binding of Sitosterol and protein was high, implying that there existed a weak binding effect. Comprehending the solvent-accessible surface area (SASA) provides valuable insights into the extent to which the complex can interact with the surrounding aqueous solution. A greater surface area implies a higher level of interaction between the complex and the aqueous solution.

Furthermore, the variation in SASA is influenced by the alterations in both the visible and hidden areas of the protein surface. The protein systems examined in this study are distinct, thus making the comparative analysis of SASA size of no significance. However, based on the fluctuation analysis of SASA, we can see that the KRAS–crystal_ligand, AKT1–crystal_ligand, MTOR–crystal_ligand, AKT1–Hesperetin, AKT1–Sitosterol, KRAS–Hesperetin, KRAS–Naringenin, MTOR–Hesperetin, and MTOR–Sitosterol systems fluctuated smoothly, indicating that the visible and hidden areas of the surfaces of these complexes undergo little change and bind better.

### 3.7. MM-GBSA Binding Free Energy

Using the trajectories from MD simulations, we utilized the MM-GBSA approach to compute the binding energy. This method provides a more precise illustration of the interaction between small molecules and target proteins. The AKT1–Hesperetin, AKT1–Sitosterol, KRAS–Hesperetin, KRAS–Naringenin, MTOR–Hesperetin, and MTOR–Sitosterol complexes had binding energies of −27.52, −47.98, −86.01, −26.42, −41.51, −23.44, −6.81, −32.21, and −37.57 kcal/mol, respectively (Table 2). A tendency for these molecules to bind to the target protein is indicated by negative values, with stronger binding being indicated by lower values. It is evident from our calculations that KRAS/Naringenin exhibits a weak binding effect, while the remaining small molecules retain the capability to bind to the corresponding proteins. It is evident from energy decomposition that van der Waals and electrostatic energies make the most significant contributions to the binding of these complexes, with the subsequent contribution being of the free energy of non-polar solvation.

## 4. Discussion

Fatigue, which is defined as difficulty in initiating or maintaining voluntary activities, is a complex physiological and pathological condition that can be categorized into two main types: peripheral fatigue and central fatigue [47,48]. Physical fatigue is an alternative term used for peripheral fatigue, which is characterized by a decline in the physical ability to perform various tasks [49,50]. Fatigue has evolved into a serious public health concern that negatively impacts human health, productivity at work, and quality of life, and about 18.3–27% of the general population is affected by fatigue [51,52]. Currently, there is a lack of effective interventions for the management of physical fatigue. Studies have shown that natural plant extracts, nutraceutical products, and active components found in natural foods can alleviate physical fatigue, suggesting that potential anti-fatigue properties are possessed by these compounds [14,53]. Nevertheless, the underlying mechanisms, targets, and pathways by which these natural compounds may mitigate physical fatigue need to be explored. Herbal medicines, particularly natural drugs, provide a substantial resource for evaluating natural compounds with significant properties and fewer adverse effects, thereby introducing an innovative approach to modern pharmaceutical exploration [7]. In the context of artificial intelligence and big data, network pharmacology (NP) is a cutting-edge technological approach used in systematic drug research. Research in this domain investigates the fundamental links that exist between diseases and syndromes at the molecular level, placing particular emphasis on the crucial pharmacodynamic constituents found in formulations of traditional Chinese medicine and the underlying mechanisms by which they exert their effects. This is achieved by evaluating the association between disease-related molecular networks and the target network of chemical compounds found in prescription drugs [54]. Network pharmacology offers novel data support for rational clinical drug use and Chinese medicine’s new drug research and development [55]. The conventional approach of “one disease-one target-one drug” in Western medicine encounters challenges in developing effective therapeutic solutions for complex or multifactorial diseases due to the multifaceted nature of diseases. TCMs exhibit promising clinical outcomes in the treatment of complex diseases through the utilization of multicomponent, multitarget, and multi-pathway mechanisms, all of which are guided by TCM theory and produce holistic effects against complex diseases [56]. Therefore, we adopted the NP technique along with molecular docking and molecular dynamics (MD) modeling to investigate the bioactive compounds present in *Citrus reticulata* peel (CR-P), as well as its potential targets and the molecular mechanism involved in treating physical fatigue. 

The present study screened and recognized Sitosterol, Naringenin, Hesperetin, Citromitin, and Nobiletin as the main bioactive compounds in CR-P by network pharmacology. It has been reported that the fluidity of the interior mitochondrial membrane increased by Sitosterol while having no effect on the outer mitochondrial membrane fluidity, thereby increasing the potential of the mitochondrial membrane and mitochondrial ATP content [57]. Possible predisposing factors in developing physical fatigue include dysfunctions in mitochondrial fluidity and energy metabolism, including fatty acid metabolism and ATP production [58]. Therefore, enhancing energy metabolism and mitochondrial fluidity may alleviate physical fatigue. Naringenin has been previously reported to enhance endurance exercise capacity and exercise duration, thereby increasing the resistance of skeletal muscle to fatigue [59]. Previous studies suggested that low physical performance and mobility are associated with mitochondrial dysfunction, reduced ATP production, and diminished muscle quality. The administration of Hesperetin resulted in a notable rise of 33% in intracellular ATP levels and a 25% increase in mitochondrial spare capacity [60], indicating that Hesperetin has the potential to enhance physical performance while coping with physical fatigue. Patients affected by liver disease frequently experience fatigue, a manifestation that profoundly affects their overall well-being [61]. The flavonoid Citromitin has been shown to have hepatoprotective effects [62]; hence, it may be involved in the process of reducing fatigue. Elevated levels and accumulation of reactive oxygen species (ROS) in the skeletal muscle are crucial contributors to the onset of physical fatigue [63]. By inhibiting ROS production and reducing superoxide dismutase (SOD) activity, Nobiletin, an antioxidant, thereby alleviates physical fatigue [64]. The efficacy of these compounds in alleviating physical fatigue was further verified and supported by molecular docking and simulation-based molecular dynamics analyses. These compounds may play a significant role in alleviating physical fatigue by acting on various targets and pathways.

Next, a network of protein–protein interactions (PPI) of 56 main overlapping targets of CR-P and physical fatigue was generated. The top ten core targets identified in the PPI network were AKT1, MTOR, KRAS, JAK2, EGFR, ESR1, HRAS, TP53, STAT3, and IL6. The potential therapeutic effects of bioactive compounds of CR-P against physical fatigue may be significantly influenced by these hub targets. Our analyses identified AKT1 as having the greatest degree of node. AKT1 is crucial in various normal and pathological cellular processes, and a lack of AKT1 leads to an elevation in the consumption of energy [65]. The significance of AKT1 as a main target for addressing fatigue generated by cancer was demonstrated in a randomized controlled trial and an in vivo study [66,67]. Moreover, AKT1 was found to be the core target by which resveratrol may act an as anti-fatigue agent in an in vitro study [68]. Among the hub targets, MTOR was the second important target in our analyses, followed by KRAS. MTOR serves as a significant modulator of cellular growth and proliferation. Phosphorylation of the intracellular Akt protein may stimulate the activation of its downstream MTOR, thereby facilitating protein synthesis [69]. As an important regulator of energy metabolism, the production of ATP is enhanced by the activation of MTOR [70]. Skeletal muscle atrophy is characterized by muscle weakness, shrinkage, and a decreased fiber cross-section area and muscle mass. It emerges as a decline in force generation that ultimately leads to physical fatigue. Understanding the intricate connection between the MTOR signaling pathway and the synthetic response of skeletal muscles is crucial in comprehending the role it plays in skeletal muscle atrophy when its activity is suppressed [71]. KRAS is a signal transducer protein that regulates cell proliferation and plays a crucial role in various cellular signaling processes. Upon receiving an upstream stimulus, it transmits activating signals to various cellular signaling pathways, such as the mitogen-activated protein kinase (MAPK) pathway, making it a critical point in the cell circuitry [72], whereas biogenesis, angiogenesis, and muscle hypertrophy are all mediated by the MAPK signaling pathway [73], making the muscle more resistant to physical fatigue. 

Furthermore, the findings of Gene Ontology (GO) enrichment analysis revealed that the prominent biological process (BP) terms, including glial cell differentiation, gliogenesis, and regulation of inflammatory response, were related to the effects of CR-P in ameliorating physical fatigue. Cellular components (CC) include blood microparticle, membrane raft, and membrane microdomain. The molecular functions (MF) mainly include icosatetraenoic acid binding, activities of transmembrane receptor protein tyrosine kinase, and antioxidants. According to the KEGG analysis, the amelioration of physical fatigue by bioactive compounds of CR-P involves multiple signaling pathways. The main signaling pathways include Phosphoinositide 3-Kinase/Protein Kinase B (PI3K-AKT), Mitogen-Activated Protein Kinase (MAPK), and Janus Kinase-Signal Transducer and Activator of Transcription (JAK-STAT) signaling pathways. A previous study suggested a potential correlation between exercise-induced fatigue and altered energy metabolism, which could be attributed to the deregulation of the PI3K-Akt signaling pathway. The modulation of myogenic cell proliferation and differentiation, as well as the growth of skeletal muscle, can be facilitated by the PI3K-AKT signaling pathway [69]. An animal study has suggested that the anti-fatigue impact of ginsenoside Rb1 in aged rats with postoperative fatigue syndrome is mediated by triggering the PI3K-AKT signaling pathway [74]. MAPK, another crucial signaling pathway, has been demonstrated to facilitate muscle repair mechanisms in both humans and animals [75]. Several studies have demonstrated the significant contribution of the MAPK signaling pathway in the transition of myoblasts from proliferation to differentiation during various stages of muscle development. Additionally, this pathway has been found to have a regulatory role in skeletal muscle function [75]. The JAK-STAT signaling cascade has been identified as having a significant role in various cell types and tissues, such as skeletal muscle. Numerous studies have highlighted the significance of the JAK/STAT pathway in controlling the myogenic progression of adult satellite cells. These cells are crucial for the postnatal growth and repair of skeletal muscle following injury.

In order to further validate the findings, further investigations involving molecular docking and MD modeling were conducted. The findings of the molecular docking analysis showed that the five bioactive compounds, including Sitosterol, Naringenin, Hesperetin, Citromitin, and Nobiletin, showed effective binding affinity with the top 10 hub targets ranging from −10.90 to −5.80 kcal/mol. The binding affinities of AKT1, MTOR, and KRAS were the lowest among the 10 targets. Binding energies < 0 kJ/mol imply spontaneous binding, whereas values of −5.0 kJ/mol or less indicate favorable binding activity [26]. Based on the binding scores, Hesperetin, Sitosterol, and Naringenin were docked with AKT1, KRAS, and MTOR proteins. As indicated by the docking results, these bioactive compounds are bound efficiently to the active sites of the hub targets; this suggests that these compounds may utilize these potential targets to mitigate physical fatigue. To further examine the interaction between AKT1 (3o90)-Sitosterol, MTOR (5gpg)-Sitosterol, AKT1 (3o90)-Hesperetin, MTOR (5gpg)-Hesperetin, KRAS (3gft)-Naringenin, and KRAS (3gft)- Hesperetin, simulations based on molecular dynamics were conducted. The interaction between the AKT1 and Hesperetin was observed to occur via amino acid residues, namely ASN-54, THR-211, and ILE-290, which formed three hydrogen bonds, whereas AKT1 interacted with Sitosterol through SER-205 and formed one hydrogen bond. Hesperetin formed six hydrogen bonds with the KRAS protein through ASP-33, GLU-31, ASP-30, ALA-146, LYS-117, and ASN-116 amino acid residues. The KRAS protein formed seven hydrogen bonds and interacted with Naringenin via THR-148, ARG-149, GLN-22, and ASN-26 amino acid residues. Similarly, Hesperetin formed two hydrogen bonds with the MTOR protein through TYR-2104 and THR-2098 amino acid residues, whereas MTOR interacted with Sitosterol via LYS-170, ASP-146, and THR-2098 amino acid residues to form four hydrogen bonds. The effective binding and interactions of these compounds with the AKT1, KRAS, and MTOR proteins are evident from hydrogen bonding analysis, where these compounds interact with different amino acids by forming hydrogen bonds. Hydrogen bonds play a crucial role in stabilizing a protein–ligand complex by forming highly specific interactions between a ligand and a receptor. They play an essential role in drug specificity, metabolism, and drug absorption in drug design [76]. Moreover, the protein–ligand complexes exhibited remarkable structural stability at a temperature of 298.15 K. Most amino acids should exhibit an RMSF value of less than 2 Å in optimal circumstances. Certain amino acids usually exhibit a higher RMSF than other amino acid residues [77]. Similarly, in our analysis, some amino acids showed higher RMSF values. However, most of the AKT1, KRAS, and MTOR regions were less than 2 Å, which indicates that the structural stability of these proteins is more apparent in the state of binding small molecules. The potential therapeutic contributions of Sitosterol and Hesperetin to CR-P in the treatment of physical fatigue were indicated by their high binding activity to the targets. However, further investigation involving in vivo and in vitro trials is necessary to validate these predictions.

There are some limitations to our investigation. Initially, the data utilized for analysis were retrieved from various databases and the literature; therefore, the quality of the data determines the credibility and precision of the predictions. Second, network pharmacology and molecular modeling are limited computer-based techniques in regard to completely assessing the precise role and comprehending the fundamental mechanism by which these bioactive compounds alleviate physical fatigue. Consequently, further validation of these findings requires in vivo and in vitro trials. 

## 5. Conclusions

The current investigation explored the bioactive compounds, potential targets, and pathways of *Citrus reticulata*-Peel (CR-P) in addressing physical fatigue through the utilization of network pharmacology, molecular docking, and molecular dynamics modeling techniques. Five bioactive compounds were screened for CR-P. A total of 56 key overlapping targets were identified between CR-P and physical fatigue, whereas the top ten hub targets in the network, including AKT1, TP53, STAT3, MTOR, KRAS, HRAS, JAK2, IL6, EGFR, and ESR1, were found, respectively. Our analysis revealed that multiple targets and pathways are involved that may play a crucial role in alleviating physical fatigue by CR-P. Bioactive compounds of CR-P, particularly Hesperetin and Sitosterol, might ameliorate physical fatigue via the PI3K-AKT signaling pathway by targeting AKT1, KRAS, and MTOR proteins. Furthermore, the MAPK signaling pathway and the JAK-STAT signaling pathway might have significant roles in the process. This study offered valuable insights into the therapy approaches for physical fatigue while providing evidence for further studies. Nevertheless, the present work provides a basis for subsequent in vitro and in vivo trials. Additional studies are required to confirm the bioactive compounds, important targets, and crucial pathways that have been identified in the present study. 

## Figures and Tables

**Table 1:** Bioactive compounds in CR-P.

ID	Molecule Name	Molecular Formula	Molecular Weight (g/mol)	OB (%)	DL
MOL000359	Sitosterol	C_29_H_50_O	414.7	36.91	0.75
MOL004328	Naringenin	C_15_H_12_O_5_	272.25	59.29	0.21
MOL005100	Hesperetin	C_16_H_14_O_6_	302.28	47.74	0.27
MOL005815	Citromitin	C_21_H_24_O_8_	404.4	86.90	0.51
MOL005828	Nobiletin	C_21_H_22_O_8_	402.4	61.67	0.52

**Table 2:** Binding free energies of protein–ligand complexes.

System Name	Δ*E*_vdw_	Δ*E*_elec_	ΔG_GB_	ΔG_SA_	ΔG_bind_
KRAS/crystal_ligand	−40.32	−2.08	20.96	−6.09	−27.52
AKT1/crystal_ligand	−89.74	5.09	45.37	−8.69	−47.98
MTOR/crystal_ligand	−92.13	−66.12	84.56	−12.32	−86.01
AKT1/Hesperetin	−38.57	−21.05	38.45	−5.25	−26.42
AKT1/Sitosterol	−63.44	−2.26	31.75	−7.56	−41.51
KRAS/Hesperetin	−31.51	−25.38	37.85	−4.40	−23.44
KRAS/Naringenin	−12.45	−6.64	13.90	−1.62	−6.81
MTOR/Hesperetin	−37.53	−45.93	56.51	−5.27	−32.21
MTOR/Sitosterol	−47.54	−2.51	18.46	−5.98	−37.57

Δ*E*_vdw_: van der Waals energy. Δ*E*_elec_: electrostatic energy. ΔG_GB_: electrostatic contribution to solvation. ΔG_SA_: non-polar contribution to solvation. ΔG_bind_: binding free energy.

## Data Availability

The data used in this research can be obtained upon reasonable request from the corresponding author.
